# Clinically and Microbiologically Derived Azithromycin Susceptibility Breakpoints for Salmonella enterica Serovars Typhi and Paratyphi A

**DOI:** 10.1128/AAC.04729-14

**Published:** 2015-04-10

**Authors:** Christopher M. Parry, Nga Tran Vu Thieu, Christiane Dolecek, Abhilasha Karkey, Ruchi Gupta, Paul Turner, David Dance, Rapeephan R. Maude, Vinh Ha, Chinh Nguyen Tran, Phuong Le Thi, Bay Pham Van Be, La Tran Thi Phi, Rang Nguyen Ngoc, Aniruddha Ghose, Sabina Dongol, James I. Campbell, Duy Pham Thanh, Tuyen Ha Thanh, Catrin E. Moore, Soeng Sona, Rajni Gaind, Monorama Deb, Ho Van Anh, Sach Nguyen Van, Hien Tran Tinh, Nicholas P. J. Day, Arjen Dondorp, Guy Thwaites, Mohamed Abul Faiz, Rattanaphone Phetsouvanh, Paul Newton, Buddha Basnyat, Jeremy J. Farrar, Stephen Baker

**Affiliations:** a Wellcome Trust Major Overseas Programme, The Hospital for Tropical Diseases, Oxford University Clinical Research Unit, Ho Chi Minh City, Vietnam; b Centre for Tropical Medicine and Global Health, Old Road Campus, University of Oxford, Oxford, United Kingdom; c Liverpool School of Tropical Medicine, Liverpool, United Kingdom; d Oxford University Clinical Research Unit, Patan Academy of Health Sciences, Kathmandu, Nepal; e Department of Microbiology, Vardhman Mahavir Medical College and Safdarjung Hospital, New Delhi, India; f Cambodia-Oxford Research Unit, Angkor Hospital for Children, Siem Reap, Cambodia; g Shoklo Malaria Research Unit, Mae Sot, Thailand; h Lao-Oxford-Mahosot Hospital-Wellcome Trust Research Unit, Mahosot Hospital, Vientiane, Laos; i Mahidol-Oxford Tropical Medicine Research Unit (MORU), Faculty of Tropical Medicine, Mahidol University, Bangkok, Thailand; j The Hospital for Tropical Diseases, Ho Chi Minh City, Vietnam; k Dong Thap Provincial Hospital, Cao Lanh, Dong Thap Province, Vietnam; l An Giang Provincial Hospital, Long Xuyen, Vietnam; m Chittagong Medical College Hospital, Chittagong, Bangladesh; n Centre for Specialized Care and Research, Chittagong, Bangladesh; o London School of Hygiene and Tropical Medicine, London, United Kingdom; p School of Tropical Medicine and Global Health, Nagasaki University, Nagasaki, Japan

## Abstract

Azithromycin is an effective treatment for uncomplicated infections with Salmonella enterica serovar Typhi and serovar Paratyphi A (enteric fever), but there are no clinically validated MIC and disk zone size interpretative guidelines. We studied individual patient data from three randomized controlled trials (RCTs) of antimicrobial treatment in enteric fever in Vietnam, with azithromycin used in one treatment arm, to determine the relationship between azithromycin treatment response and the azithromycin MIC of the infecting isolate. We additionally compared the azithromycin MIC and the disk susceptibility zone sizes of 1,640 *S*. Typhi and *S*. Paratyphi A clinical isolates collected from seven Asian countries. In the RCTs, 214 patients who were treated with azithromycin at a dose of 10 to 20 mg/ml for 5 to 7 days were analyzed. Treatment was successful in 195 of 214 (91%) patients, with no significant difference in response (cure rate, fever clearance time) with MICs ranging from 4 to 16 μg/ml. The proportion of Asian enteric fever isolates with an MIC of ≤16 μg/ml was 1,452/1,460 (99.5%; 95% confidence interval [CI], 98.9 to 99.7) for *S*. Typhi and 207/240 (86.3%; 95% CI, 81.2 to 90.3) (*P* < 0.001) for *S*. Paratyphi A. A zone size of ≥13 mm to a 5-μg azithromycin disk identified *S*. Typhi isolates with an MIC of ≤16 μg/ml with a sensitivity of 99.7%. An azithromycin MIC of ≤16 μg/ml or disk inhibition zone size of ≥13 mm enabled the detection of susceptible *S*. Typhi isolates that respond to azithromycin treatment. Further work is needed to define the response to treatment in *S*. Typhi isolates with an azithromycin MIC of >16 μg/ml and to determine MIC and disk breakpoints for *S*. Paratyphi A.

## INTRODUCTION

Enteric fever, caused by Salmonella enterica serovars Typhi and Paratyphi A, is common among febrile patients in regions of the world that have poor standards of hygiene and sanitation. It has been estimated that there may be as many as 27 million new infections of enteric fever each year (1). Although the disease can be treated and complications can be prevented by the use of appropriate antimicrobials, antimicrobial-resistant strains of *S*. Typhi and *S*. Paratyphi A have become common in regions of endemicity, which has made treatment selection a challenge (2). Multidrug-resistant (MDR) strains (exhibiting resistance to chloramphenicol, trimethoprim-sulfamethoxazole, and ampicillin) and those with intermediate susceptibility or resistance to fluoroquinolones such as ciprofloxacin and ofloxacin are now widespread in Asia and Africa (3–6). Extended-spectrum cephalosporins such as ceftriaxone and cefixime are commonly used for infections caused by MDR organisms and in children, although these tend to be associated with slower fever clearance times (FCTs), and sporadic reports of extended-spectrum beta-lactamase (ESBL)-producing isolates are a concern (7).

Several randomized clinical trials (RCTs) have established the azalide antimicrobial azithromycin to be an effective alternative oral treatment for uncomplicated enteric fever. Treatment durations of 5 to 7 days lead to the resolution of symptoms with generally low rates of relapse and convalescent-stage fecal carriage (8–14). Given the trends of antimicrobial resistance in enteric fever, azithromycin is likely to become one of the few universally efficacious antimicrobials for treating the disease. Therefore, the laboratory detection and identification of strains with decreased susceptibility or resistance to azithromycin is important for clinicians treating individual patients and for public health organizations setting routine treatment guidelines. Current guidelines have no clinically validated interpretive ranges for azithromycin MICs or disk susceptibility breakpoints for Salmonella isolates. The epidemiological surveillance of bacterial populations has led to the recommendation that an azithromycin MIC of ≤16 μg/ml be considered susceptible for invasive isolates of Salmonella (15–17).

To address these knowledge gaps in the use of azithromycin for treating enteric fever, we aimed to examine the relationship between the MIC against azithromycin of infecting isolates and the clinical response to azithromycin in adults and children recruited to three RCTs of enteric fever conducted in Vietnam. We additionally aimed to study the relationship between azithromycin MIC distribution and disk inhibition zone size in over 1,500 clinical isolates of *S*. Typhi and *S*. Paratyphi A from seven Asian countries. Using these data, we propose evidence-derived MIC and disk susceptibility test breakpoints for azithromycin treatment in enteric fever.

## MATERIALS AND METHODS

### Ethics statement.

The study was conducted according to the principles expressed in the Declaration of Helsinki. The RCTs on which the data for this study were derived were approved by the Institutional Review Board of the Hospital for Tropical Diseases and the additional hospitals involved in the studies. All patients in the clinical trials provided informed consent (informed consent was provided by the parents or guardian of children under 18 years of age) for the collection of samples and subsequent analysis.

### Salmonella Typhi and Salmonella Paratyphi A strain collection.

The Salmonella Typhi and Salmonella Paratyphi A strains used in this study were isolates collected from blood culture, and occasionally from bone marrow or fecal culture, as part of the routine diagnostic activities of microbiology laboratories in seven countries. The participating laboratories were the following: The Hospital for Tropical Diseases, Ho Chi Minh City, Vietnam; Dong Thap Provincial Hospital, Dong Thap Province, Vietnam; An Giang Provincial Hospital, An Giang Province, Vietnam; Angkor Hospital for Children, Siem Reap, Cambodia; Mahosot Hospital, Vientiane, Laos; Shoklo Malaria Research Unit, Mae Sot, Thailand; Chittagong Medical College Hospital, Chittagong, Bangladesh; Patan Hospital, Kathmandu, Nepal; and Safdarjung Hospital, New Delhi, India. Only one isolate (the strain isolated on admission to the health care facility) from each patient was included for microbiological examination and analysis.

### Microbiological methods.

The isolates were identified by standard biochemical tests and agglutination with Salmonella-specific antisera. Antimicrobial susceptibility tests were performed at the time of isolation by the modified Kirby-Bauer disk diffusion method, with zone sizes measured and recorded. Zone size interpretation was based on the 2013 CLSI guidelines (15). The antimicrobial disks tested were chloramphenicol (CHL; 30 μg), trimethoprim-sulfamethoxazole (SXT; 1.25 and 23.75 μg), ampicillin (AMP; 10 μg), ceftriaxone (CRO; 30 μg), ofloxacin (OFX; 5 μg), ciprofloxacin (CIP; 5 µg), nalidixic acid (NAL; 30 μg), and azithromycin (AZM; 5 μg). An isolate was defined as MDR if it was resistant to chloramphenicol, trimethoprim-sulfamethoxazole, and ampicillin by disk susceptibility testing. An isolate was defined as having intermediate susceptibility to ciprofloxacin if it was resistant to nalidixic acid or had a ciprofloxacin MIC of 0.125 to 0.5 μg/ml and resistant if the ciprofloxacin MIC was ≥1.0 μg/ml.

At the time of isolation, or after a period of storage at −20°C or −80°C, the MICs of the isolates were determined by the standard agar plate dilution method according to CLSI guidelines with a targeted final inoculum of 5 × 10^5^ CFU/ml (18) or by Etest, according to manufacturer's recommendations (AB Biodisk, Sweden). Azithromycin powder for the agar plate dilution MICs was a gift from Pfizer, United Kingdom. Escherichia coli ATCC 25922 and Staphylococcus aureus ATCC 29213 were used as control strains for these assays.

### Analysis of isolates for macrolide resistance genes.

The presence of macrolide resistance genes was determined in available isolates that had an elevated MIC to azithromycin (≥16 μg/ml) and/or a decreased zone of inhibition, ≤18 mm, to an azithromycin 5-μg disk. Genomic DNA was extracted using the Wizard Genomic DNA kit (Promega, Madison, WI) as per the manufacturer's instruction and investigated by PCR amplification to detect *mphA*, *ermA*, *ermB*, *ermV*, *ereA*, *ereB*, *mefA*, and *msrA* genes using published methods (19). All PCRs included positive and negative controls.

### Randomized controlled trials.

We analyzed the results of three open RCTs conducted in southern Vietnam between 1997 and 2005, in which azithromycin was used for the treatment of enteric fever in one of the trial arms (10, 13, 14). All the RCTs were conducted using a standard protocol, except for the dose and duration of azithromycin treatment and the alternative treatment regimens used. The RCTs were conducted at three study sites in southern Vietnam: The Hospital for Tropical Diseases, Ho Chi Minh City (10, 14); Dong Thap Provincial Hospital, Cao Lanh, Dong Thap Province (13, 14); and An Giang Provincial Hospital, Long Xuyen, An Giang Province (14).

### Clinical procedures.

Patients with suspected uncomplicated enteric fever were allocated to one of each of the treatment groups in an open randomized comparison. A computer-generated randomization list was produced by an administrator who was not otherwise involved in the trial. The treatment allocations were kept in serially numbered sealed opaque envelopes that were opened only after the patient had been enrolled into the study. The treatment arms were azithromycin (Zithromax suspension, 200 mg/5 ml; or Zithromax tablets, 500 mg/tablet; both from Pfizer, USA) at a dose that varied between 10 and 20 mg/kg of body weight/day orally in a single daily dose (maximum, 1 g daily) for 5 days (10) or 7 days (13, 14). The comparator arms were ofloxacin (10, 13), a combination of ofloxacin and azithromycin (13), or gatifloxacin (14). Hematocrit, white cell, platelet, and blood differential counts were performed with serum aspartate transaminase, alanine transaminase, creatinine levels, and urinalysis before therapy was initiated. Aspartate transaminase and alanine transaminase measurements were repeated 1 day after the end of therapy. A full blood count was repeated if there was a suggestion of gastrointestinal bleeding or clinical evidence of anemia.

Patients were excluded if they refused consent, had evidence of worsening or complicated disease, had inability to swallow oral medication, had a history of significant underlying disease, had hypersensitivity to either of the trial drugs, or were pregnant or lactating. Additionally, patients who gave a history of treatment with a fluoroquinolone, a third-generation cephalosporin, or a macrolide within 1 week of hospital admission were also excluded.

### Clinical definitions.

In all three studies, patients were examined daily, with axillary temperature measured every 6 h, until discharge from hospital, with particular reference to clinical symptoms and complications of the disease. Response to treatment was assessed by the resolution of clinical symptoms and signs, the fever clearance time (time from the start of treatment until the axillary body temperature reached ≤37.5°C and remained at this temperature for at least 48 h), the development of complications or death, any evidence of relapse of infection, and persistent fecal carriage after the conclusion of treatment or at the 1-month follow-up visit.

Clinical treatment failure was defined as the persistence of fever (>37.5°C) and other enteric fever-related symptoms for more than 2 days after the end of treatment or the development of severe complications (severe gastrointestinal bleeding, intestinal perforation, visible jaundice, myocarditis, pneumonia, renal failure, shock, or an altered consciousness level, i.e., with a Glasgow coma score [GCS] of <15/15) during treatment and the need for rescue treatment in the judgment of the treating clinician. Microbiological treatment failure was defined as isolation of Salmonella Typhi or Salmonella Paratyphi A from blood or a sterile site after the completion of treatment. Poststudy fecal carriage was defined as a positive fecal culture, with an isolate having the same susceptibility pattern as the original isolate, after the end of the initial treatment and before hospital discharge.

Patients were requested to return for a follow-up assessment at 4 weeks or earlier if their symptoms recurred, and then at 3 and 6 months. Clinical evidence of relapse was sought, and one fecal culture was performed. A blood culture was performed if the symptoms and signs suggested relapse. A relapse was defined as a recurrence of symptoms and signs suggestive of enteric fever within the 4-week period after the patient had been discharged as healthy from the hospital accompanied by a blood culture positive for Salmonella Typhi or Salmonella Paratyphi A. Fecal carriage was defined as a positive fecal culture at a follow-up visit, with an isolate having the same susceptibility pattern as the original isolate.

### Statistical analysis.

Analysis of the RCTs was restricted to patients in whom *S*. Typhi or *S*. Paratyphi A was isolated from blood or bone marrow culture prior to treatment with azithromycin and for whom the azithromycin MIC of the original infecting isolate had been determined. The pooled admission and outcome data for individual patients were compiled with respect to the azithromycin MIC of the original infecting isolate. Proportions were compared with the chi-square test, Fisher's exact test, or analysis of variance. Normally distributed data were compared using the Student *t* test, and nonnormally distributed data were compared using the Mann-Whitney U test or the Kruskal-Wallis test. The fever clearance times were compared using survival analysis and the log rank test. Independent risk factors for clinical failure in the clinical trials were determined by multivariate logistic regression; a *P* value of <0.05 was considered significant. Statistical analysis was performed using SPSS for Windows version 21 (SPSS Inc., Chicago, IL, USA).

For the microbiology data, an MIC histogram was constructed, and the MIC_50_ and MIC_90_ values were calculated. The disk zone diameter breakpoints were selected by the modified error rate-bounded method of Metzler and DeHaan and adjusted until the number of very major (false-susceptible) and major (false-resistant) errors had been minimized (20). The proposed MIC breakpoints for susceptibility based on the clinical data were ≤16 μg/ml. Guidelines for acceptable discrepancy rates were according to the CLSI recommendation (21). Because of the inherent ±1 dilution variation in MIC testing for each serovar, discrepancy rates were calculated for the susceptible MIC − 1 dilution, the susceptible and resistant MICs, and the resistant MIC + 1 dilution. For the zone size interpretive criteria, the following acceptable discrepancy rates have been established by the CLSI: for R + S, 10% very major and 10% major discrepancies; for R + 1, 2% very major discrepancies; and for S − 1, 2% major discrepancies (21).

## RESULTS

### Analysis of randomized controlled trials.

There were 248 culture-positive patients randomized to azithromycin in the three trials. In 34 of these patients, the bacterial isolate was not available for rechecking the azithromycin susceptibility pattern, leaving 214 patients eligible for analysis. This final data set of 214 patients had a median age of 13 years (interquartile range [IQR], 8 to 20; range, 1 to 68) and a median duration of illness prior to admission of 8 days (IQR, 6 to 10; range, 2 to 30). The infecting isolate was *S*. Typhi in 209 patients and *S*. Paratyphi A in 5 patients. A total of 137 (64%) isolates were MDR, 184 (86%) were intermediate to ciprofloxacin, and none were ciprofloxacin resistant. All isolates were susceptible to ceftriaxone. The median azithromycin MIC was 8 μg/ml (IQR, 8 to 12; range, 4 to 16). The demographic, clinical, and microbiological features of the patients grouped by the azithromycin MIC are shown in Table 1. There were no significant differences between the three groups, but isolates with an MIC of 12 to 16 μg/ml were more likely to be isolated from patients in the studies conducted in the Mekong Delta (13, 14).

**TABLE 1:** Demographic, clinical, and microbiological features of patients with uncomplicated enteric fever treated with azithromycin from three randomized trials

Variable	Value^*a*^ for patients with infecting isolate azithromycin MIC of:			*P* value
	4 μg/ml	6–8 μg/ml	12–16 μg/ml	
No. of patients	13	116	85	
Age (yr)	14 (9–17)	14 (9–21)	11 (8–19)	0.256
Male sex (%)	8 (61.5)	59 (50.9)	35 (41.2)	0.233
Days of illness (IQR)	9 (6–14)	8 (6–10)	7 (5–10)	0.078
Patients from Dong Thap/An Giang (%)	8 (61)	78 (73)	79 (93)	<0.001
Patients from HCMC^*b*^ (%)	5 (39)	31 (27)	6 (7)	
Headache (%)	9 (69)	31 (75)	58 (68)	0.582
Cough (%)	3 (23)	28 (24)	27 (32)	0.428
Vomiting (%)	5 (39)	41 (35)	31 (37)	0.969
Abdominal pain (%)	4 (31)	55 (47)	43 (51)	0.410
Constipation (%)	1 (8)	12 (10)	24 (29)	0.002
Diarrhea (%)	8 (62)	86 (74)	54 (64)	0.227
Antimicrobial pretreatment (%)	1 (8)	20 (17)	10 (12)	0.252
Admission temp (°C)	39.5 (39.0–39.9)	39.0 (38.9–40.0)	39.0 (38.5–39.5)	0.677
Hepatomegaly (%)	7 (54)	47 (41)	41 (48)	0.431
Splenomegaly (%)	1 (8)	9 (8)	7 (8)	0.994
Hematocrit (%)	38 (34–40)	37 (32–40)	34 (31–38)	0.041
White cell count (×10^9^/liter)	7.7 (5.5–9.2)	6.8 (5.0–8.3)	7.2 (5.5–8.8)	0.326
Neutrophil (%)	72 (63–79)	66 (55–73)	67 (58–76)	0.316
Lymphocytes (%)	19 (15–35)	29 (20–37)	26 (19–35)	0.378
Platelets (×10^9^/liter)	213 (187–270)	166 (120–213)	175 (140–259)	0.004
AST (IU/liter)	154 (68–202)	77 (44–131)	96 (60–145)	0.065
ALT (IU/liter)	100 (38–221)	63 (40–103)	69 (43–127)	0.207
*S*. Typhi (%)	13 (6)	115 (55)	81 (39)	0.173
*S*. Paratyphi A (%)	0 (0)	1 (20)	4 (80)	
MDR isolate^*c*^ (%)	7 (54)	78 (67)	52 (61)	0.495
Ciprofloxacin intermediate (%)	8 (62)	102 (88)	74 (87)	0.032

a Values are medians (IQR) of given unit or numbers (%).

b HCMC, The Hospital for Tropical Diseases, Ho Chi Minh City, Vietnam.

c MDR, multidrug resistant (resistant to chloramphenicol, ampicillin, and trimethoprim-sulfamethoxazole).

**FIG 1:**
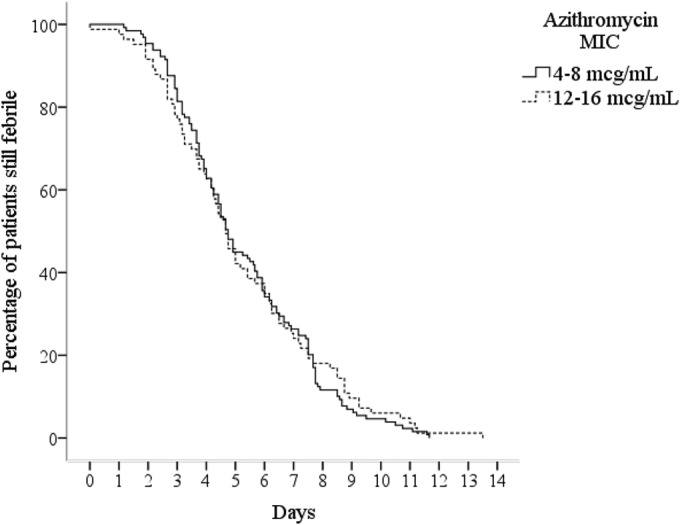
Clinical response to azithromycin in the treatment of enteric fever by fever clearance time. Kaplan-Meier curves show the proportion of patients still febrile after starting azithromycin according to the azithromycin MIC of the infecting isolate.

The response to treatment in relation to the azithromycin MIC of the infecting isolate is shown in Table 2; 195 patients (91.1%; 95% CI, 86.3 to 94.4) successfully completed their treatment, and 19 patients failed treatment. Five treatment failures were microbiological failures with a positive blood culture after the completion of treatment, and 18 were clinical failures due to persisting fever and symptoms on the 10th day, including 10 patients who developed a complication. Some failed in more than one of these categories. The median (IQR, range) FCT was 4.8 (3.3 to 7.2, 0.5 to 13.5) days, and the median (IQR, range) duration of hospital stay was 13 (12 to 15, 9 to 26) days. A Kaplan-Meier curve analysis of the FCT found no significant difference in the clinical response to azithromycin according to the MIC of the infecting isolate (Fig. 1). Longitudinal posttreatment follow-up was possible in 180 patients as follows: on a single follow-up occasion in 23 patients, on two occasions in 66 patients, and on three occasions in 91 patients. There were no recorded relapses. Three patients had a positive fecal culture at the follow-up visit: two patients at the 1-month follow-up visit and one patient at the 3-month follow-up visit. None of these patients failed their initial course of treatment. Table 3 outlines the association between demographics, clinical observations, treatment regimen, and microbiological factors against treatment failure. We found no significant associations between the selected variables and clinical failure by either a univariate or a multivariate (data not shown) analysis.

**TABLE 2:** Clinical response to azithromycin in relation to the azithromycin MIC of the infecting isolate in enteric fever treatment^*a*^

Variable	Value for patients with infecting isolate azithromycin MIC of:			*P* value
	4 μg/ml	6–8 μg/ml	12–16 μg/ml	
No. of patients	13	116	85	
Median duration (IQR) to fever clearance time (days)	4.4 (3.7–4.5)	4.9 (3.4–7.5)	4.7 (3.2–7.0)	0.249
Any failure (%)	1 (8)	9 (8)	9 (11)	0.775
Clinical failure (%)	1 (8)	9 (8)	8 (9)	0.912
Microbiological failure (%)	1 (8)	1 (1)	3 (4)	0.195
Complicated disease (%)	0 (0)	4 (3)	6 (7)	0.347
Median duration (range) of hospital stay (days)	12 (11–14)	13 (12–15)	13 (12–15)	0.714
Convalescent-stage fecal carriage (%)	0/10 (0)	0/98 (0)	3/72 (4)	
Relapse (%)	0/10 (0)	0/98 (0)	0/72 (0)	

a Unless otherwise indicated, values are numbers (%) of patients exhibiting the response.

**TABLE 3:** Factors associated with treatment failure with azithromycin therapy for enteric fever

Variable	Value^*a*^ for treatment outcome		*P* value	OR^*b*^ (95% CI)
	Failure	Success		
No. of patients	19	195		
Male sex (%)	12 (8–22)	13 (8–20)	0.966	
Days of illness (IQR)	7 (4–14)	8 (6–10)	0.601	
Male sex (%)	10 (52.6)	92 (47.2)	0.831	1.24 (0.44–3.51)
Mekong Delta site (%)	17 (89.5)	155 (79.5)	0.379	2.19 (0.49–20.3)
*S*. Typhi (%)	19 (100)	190 (97)	1.000	
*S*. Paratyphi A (%)	0 (0)	5 (3)		
MDR isolate (%)	15 (78.9)	122 (62.6)	0.242	2.24 (0.68–9.61)
Ciprofloxacin intermediate (%)	17 (89.5)	167 (85.6)	1.00	1.43 (0.31–13.4)
Azithromycin MIC >8 μg/ml (%)	9 (47.4)	76 (39.0)	0.640	1.41 (0.50–3.97)
Duration of azithromycin treatment <7 days (%)	2 (10.5)	40 (20.5)	0.379	0.46 (0.05–2.06)
Dose of azithromycin 10 mg/kg (%)	5 (26.3)	40 (20.5)	0.559	1.38 (0.37–4.37)

a Values are medians (interquartile range) or numbers (%).

b OR, odds ratio.

### Antimicrobial susceptibility testing of *S*. Typhi and *S*. Paratyphi A isolates against azithromycin.

We analyzed the antimicrobial susceptibility profiles and measured the zone sizes and MICs against azithromycin in 1,700 invasive Salmonella isolates. These strains were isolated in seven countries across Asia and spanned 17 years; 1,460 of these were *S*. Typhi, and 240 were *S*. Paratyphi A (Table 4). For the *S*. Typhi isolates, 510/1,460 (34.9%) were MDR, 948/1,460 (64.9%) demonstrated intermediate susceptibility to ciprofloxacin, and 42/1,460 (2.9%) were ciprofloxacin resistant. For *S*. Paratyphi A isolates, 0/240 (0%) were MDR, 184/240 (76.7%) demonstrated intermediate susceptibility to ciprofloxacin, and 27/240 (11.3%) were ciprofloxacin resistant. The proportion of ciprofloxacin-nonsusceptible *S*. Paratyphi A isolates was 211/240 (87.9%), significantly higher than the proportion of *S*. Typhi isolates at 990/1,460 (67.8%) (*P* < 0.001, Fisher's exact test).

**TABLE 4:** Organisms subjected to antimicrobial susceptibility and azithromycin MIC testing in this study

Country	Serovar	No. of organisms collected in:										
		1995–2001	2004	2005	2006	2007	2008	2009	2010	2011	2012	Total
Bangladesh	Typhi										29	29
	Paratyphi										3	3
	Total										32	32
Cambodia	Typhi					19	25	14	36	50	96	240
	Paratyphi					0	3	0	0	0	0	3
	Total					19	28	14	36	50	96	243
India	Typhi					92	72	86				250
	Paratyphi					20	15	20				55
	Total					112	87	106				305
Laos	Typhi		26	16	35	21	36	32	42	18	6	232
	Paratyphi		0	0	0	0	0	1	0	0	0	1
	Total		26	16	35	21	36	33	42	18	6	233
Nepal	Typhi			47	67	21	29	4	1			379
	Paratyphi			113	109	100	47	6	4			169
	Total			160	176	121	76	10	5			548
Thailand	Typhi					22	20	2	1			45
	Paratyphi					0	0	0	0			0
	Total					22	20	2	1			45
Vietnam	Typhi	162	88	35								285
	Paratyphi	5	1	3								9
	Total	167	89	38								294
Total	Typhi	162	114	164	144	254	200	140	83	68	131	1,460
	Paratyphi	5	1	50	67	41	47	25	1	0	3	240
	Total	167	115	214	211	295	247	165	84	68	134	1,700

**FIG 2:**
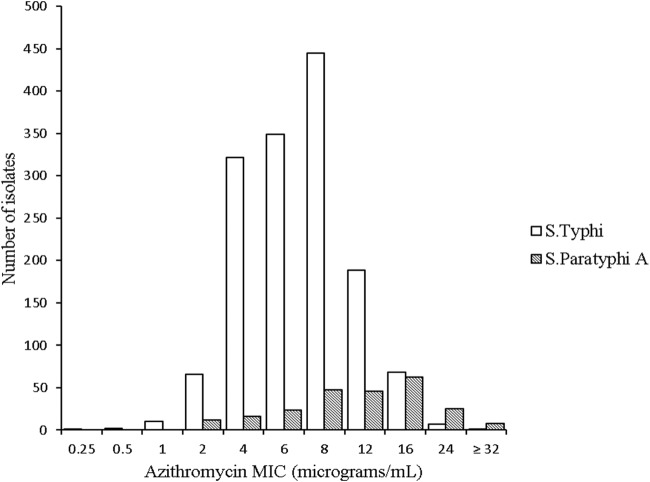
Distribution of azithromycin MICs in *S*. Typhi and *S*. Paratyphi A. Histogram showing the azithromycin MIC distribution for 1,460 *S*. Typhi isolates and 240 *S*. Paratyphi A isolates from Bangladesh (*n* = 32), Cambodia (*n* = 243), India (*n* = 305), Laos (*n* = 233), Nepal (*n* = 548), Thailand (*n* = 45), and Vietnam (*n* = 294).

The distribution of MICs against azithromycin of the 1,700 Salmonella isolates is shown in Fig. 2. The MICs against azithromycin in the *S*. Typhi isolates were normally distributed and ranged between 0.25 μg/ml and >32 μg/ml, with MIC_50_ and MIC_90_ values of 6 μg/ml and 12 μg/ml, respectively. For the *S*. Paratyphi A isolates, the MICs against azithromycin ranged from 1 μg/ml to >32 μg/ml and the corresponding MIC_50_ and MIC_90_ values were 12 μg/ml and 24 μg/ml, respectively. The proportion of *S*. Typhi isolates with an MIC of ≤16 μg/ml against azithromycin was 1,452/1,460 (99.5%; 95% CI, 98.9 to 99.7), and the corresponding proportion for the *S*. Paratyphi A isolates was 207/240 (86.3%; 95%, CI 81.2 to 90.3) (*P* < 0.001, Fisher's exact test).

**FIG 3:**
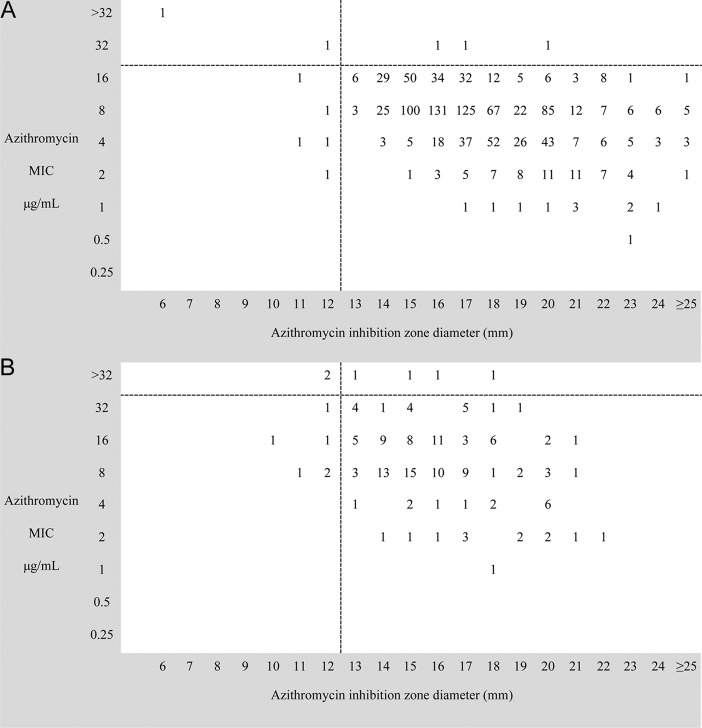
Relationship between azithromycin MIC and inhibition zone size in invasive Salmonella isolates. (A) Scatter plot of MIC data from 1,062 *S*. Typhi isolates from Bangladesh (*n* = 29), Cambodia (*n* = 240), India (*n* = 250), Nepal (*n* = 213), Thailand (*n* = 45), and Vietnam (*n* = 285). (B) Scatter plot of MIC data from 156 Salmonella serotype Paratyphi A isolates from Bangladesh (*n* = 2), Cambodia (*n* = 3), India (*n* = 52), Nepal (*n* = 90), and Vietnam (*n* = 9). Both plots show the relationship between the MIC to azithromycin (*y* axis) and the inhibition zone diameters using a 5-μg azithromycin disk.

Azithromycin disk inhibition zone sizes were available for 1,062 of the *S*. Typhi isolates and 156 of the *S*. Paratyphi A isolates. The relationships between azithromycin MIC and disk inhibition zone size for *S*. Typhi and *S*. Paratyphi A are shown in Fig. 3. There was a substantial spread of zone sizes in comparison to MICs; for example, the majority of *S*. Typhi isolates (591/1,062 [55.6%]) had an MIC of 8 μg/ml, and the corresponding zone sizes spanned 12 to 27 mm. Table 5 summarizes the proportion of false-susceptible results (very major discrepancies) and false-resistant results (major discrepancies) using an MIC breakpoint of ≤16 μg/ml and a zone size breakpoint of ≥13 mm to a 5-μg azithromycin disk for susceptibility in *S*. Typhi isolates. The numbers of very major and major errors all amounted to less than 2% and were within the CLSI guidelines (22). When ≤32 μg/ml was selected as the MIC breakpoint and a zone of inhibition ≥13 mm to a 5-μg azithromycin disk for susceptible *S*. Paratyphi A isolates, the proportion of major errors in the R + S group was 17.4% and that of major errors in the S − 1 group was 3.8%, both of which are outside the acceptable CLSI guidelines limits.

**TABLE 5:** Proportion of false-susceptible and false-resistant results for proposed azithromycin MIC breakpoints

Organism (susceptibility breakpoint [μg/ml])	MIC range^*a*^	No. of isolates	No. of discrepancies (discrepancy rate [%])	
			Very major	Major
Typhi (≤16)	≥R + 1	1	0	NA^*b*^
	R + S	191	3 (1.6)	1 (0.5)
	≤S + 1	870	NA	4 (0.5)
	Total	1,062	3 (0.3)	5 (0.5)
Paratyphi A (≤32)	≥R + 1	0	0	NA
	R + S	23	4 (17.4)	1 (4.3)
	≤S + 1	133	NA	5 (3.8)
	Total	156	4 (2.6)	6 (3.8)

a S, susceptible MIC; R, nonsusceptible MIC.

b NA, not applicable.

We hypothesized that the strains with elevated MICs to azithromycin (≥16 μg/ml and/or zone of inhibition of ≤18 mm) harbored plasmid-borne macrolide resistance genes. To investigate their presence, we extracted genomic DNA from 39 *S*. Paratyphi A isolates (Nepal [*n* = 38], Cambodia [*n* = 1]) and 40 *S*. Typhi isolates (Nepal [*n* = 14], Cambodia [*n* = 15], Laos [*n* = 6], Vietnam [*n* = 5]). Despite the amplification of the appropriate positive controls, we were unable to detect the presence of *mphA*, *ermA*, *ermB*, *ermV*, *ereA*, *ereB*, *mefA*, and *msrA* genes in these isolates.

## DISCUSSION

Interpretative breakpoints for disk susceptibility testing with antimicrobials used for treatment are necessary to assist clinicians in the choice of therapy, for the collection of accurate surveillance data, and for the detection of emerging resistance. The lack of validated guidelines for azithromycin susceptibility in *S*. Typhi and *S*. Paratyphi A is a significant problem in the clinical management of enteric fever. The continued use of azithromycin in enteric fever infections with reduced susceptibility to azithromycin may inadvertently drive the emergence and spread of azithromycin-resistant isolates and lead to treatment failure. The establishment of suitable breakpoints requires the evaluation of several sources of evidence, including clinical outcome data, MIC distributions for the pathogen, the investigation of potential resistance mechanisms, and consideration of the pharmacodynamic and pharmacokinetic properties of the antimicrobial (21).

Here we have analyzed three RCTs conducted in Vietnam. Patients with uncomplicated enteric fever treated with oral azithromycin had a pooled success rate of 91% (95% CI, 86 to 94). The trials were conducted according to similar protocols, although it should be noted that the duration and dosage of azithromycin treatment were not standard across all patient groups. The azithromycin MIC of the isolates ranged between 4 μg/ml and 16 μg/ml, which we found to be typical of strains isolated across Asia. We found no significant difference in the response to azithromycin treatment according to the MIC of the infecting organism. Furthermore, when combined with other clinical and treatment factors, we found there to be no influence of azithromycin MIC on treatment outcome. These data predict that oral azithromycin is an acceptable choice for treating adults and children with uncomplicated enteric fever, provided the azithromycin MIC of the infecting strain is ≤16 μg/ml. We recognize that the optimum dose and duration of treatment remain to be determined.

The lack of infections with isolates with an MIC of >16 μg/ml is an obvious limitation of this study, and more information on the response to treatment when patients are infected with strains with higher MICs is needed. Furthermore, we had data from only five patients from whom an *S*. Paratyphi A isolate was recovered in the clinical trial analysis, and this lack of clinical data for *S*. Paratyphi A is a further limitation. Antimicrobial-resistant *S*. Paratyphi A causes a significant burden of disease in Asia and may be increasing. Worryingly, we found that *S*. Paratyphi A isolates were significantly more likely to have an azithromycin MIC of >16 μg/ml than *S*. Typhi isolates and were additionally more likely to be ciprofloxacin nonsusceptible. Of note, in a case report of the clinical failure of azithromycin treatment in enteric fever caused by *S*. Paratyphi A, the isolate had an azithromycin MIC of 64 μg/ml initially, and then the MIC was 256 μg/ml in a second blood culture (23). In this case, the specific mechanism of this resistance was not described, and there are few reports on the mechanisms of resistance to azithromycin in Salmonella spp. Non-*S*. Typhi Salmonella strains with mutations in the *rplD* gene and containing the *mphA* gene have been described previously (24). Here we did not identify any previously described macrolide resistance genes in *S*. Paratyphi A strains with elevated MICs and/or reduced zone sizes, but we did not exhaustively search for other mechanisms. We suggest that further *in vitro* experiments be performed to understand how intrinsic increases in MIC may occur through changes in transcription or posttranslational modification.

The isolates analyzed for MIC and disk susceptibility for the purposes of this investigation were amalgamated from seven countries across Asia, making this the biggest study of its type providing evidence for azithromycin susceptibility breakpoints in enteric fever. The majority of these strains were collected in the last 10 years, although some Vietnamese isolates dated back to the mid-1990s. For *S*. Typhi isolates, the majority had MICs ranging between 4 and 12 μg/ml with only 0.5% having an MIC of >16 μg/ml. These data are consistent with previous data presented for *S*. Typhi and non-*S*. Typhi Salmonella isolates and support the concept of an ecological cutoff for wild-type *S*. Typhi of ≤16 μg/ml of azithromycin, with isolates with an MIC of >16 μg/ml being considered non-wild-type strains (24, 25). A disk zone size of ≥13 mm identified the majority of isolates with an MIC ≤16 μg/ml. The breakpoint missed some isolates with a higher MIC, although a limitation of this study is the lack of isolates with an MIC of >16 μg/ml.

The MIC distribution against azithromycin in *S*. Paratyphi A was not concordant with that of *S*. Typhi. The MICs in *S*. Paratyphi were skewed to the right, with 13.8% of isolates having an MIC of >16 μg/ml. These data suggest that the epidemiological cutoff for wild-type *S*. Paratyphi A may be 32 μg/ml, which is higher than that of the *S*. Typhi strains. A similar discordance between the azithromycin susceptibilities of *S*. Typhi and *S*. Paratyphi A was observed in a smaller study conducted in Kolkata, India (26). As with the *S*. Typhi isolates, a disk zone size of ≥13 mm identified the majority of *S*. Paratyphi A isolates with an MIC of ≤32 μg/ml but missed some isolates with a higher MIC. The proportions of very major and major errors using this disk breakpoint were not within acceptable limits as recommended by the CLSI (21). Furthermore, there were few isolates with an MIC of >32 μg/ml. The data from this study suggest that *S*. Paratyphi A and *S*. Typhi cannot be assigned to the same MIC breakpoint or zone diameter criteria. *S*. Typhi and *S*. Paratyphi A are two serovars of the same bacterial species, Salmonella enterica. The observed difference in MIC distribution between the two serovars is not consistent with the usual rule that the same species should have the same MIC distribution regardless of the serovar. MIC data from other strain collections are needed to confirm this difference.

We acknowledge that broth microdilution is the internationally recognized reference method for MIC determination (ISO 20776-1 and -2) rather than the Etest or agar dilution method, and this is a limitation. We are not aware of a study formally comparing the three methods for azithromycin MIC testing in Salmonella enterica. We further recognize that there may have been some variation in the performances of the susceptibility tests performed across the different sites, by different scientists, and using different reagent manufacturers, although all sites adhere to CLSI guidelines. Test factors such as pH and inoculum have been shown to have important effects on azithromycin susceptibility testing of Salmonella (27). Furthermore, the margin of the zone of inhibited growth around the azithromycin disk may not be clear and may be difficult to interpret accurately.

Azithromycin is an azalide antimicrobial with excellent tissue penetration (28). It achieves concentrations in macrophages and neutrophils that are >100-fold higher than those measured in serum (29, 30). The drug also has a half-life of 2 to 3 days, which allows once-a-day dosing (28). The pharmacokinetic-pharmacodynamic (PK-PD) parameters predictive for the efficacy for azithromycin in enteric fever have not been determined, but in other studies, free drug area under the concentration-time curve from 0 to 24 h/MIC ratio (AUC_24H_/MIC ratio) was the parameter found to be most predictive of efficacy (31). It may be that levels of azithromycin in plasma and an *in vitro* MIC result are not the best measures of efficacy for this antimicrobial, which is highly concentrated at the site of intracellular infection. The pharmacokinetics of azithromycin was not measured in the trials studied here, and we advocate that future trials in enteric fever must incorporate pharmacokinetic measurements to allow correct analysis of PK-PD parameters.

In summary, our data support the proposition that an azithromycin MIC of ≤16 μg/ml defines a wild-type population of *S*. Typhi isolates (24, 25). We further show that this MIC defines a population of isolates associated with a satisfactory response to azithromycin treatment in uncomplicated disease and propose tentative disk susceptibility breakpoints that will detect such isolates. We recognize that there is insufficient clinical and PK-PD data to determine the response to treatment in infections with *S*. Typhi isolates with an azithromycin MIC of >16 μg/ml or generally with *S*. Paratyphi A infections. We are aware of sporadic cases of treatment failure with *S*. Paratyphi A infections with azithromycin MIC of <16 μg/ml (22, 32) and increasing reports of isolates from enteric fever patients with an azithromycin MIC of >16 μg/ml (33, 34). We suspect that the use of azithromycin to treat enteric fever may be driving their emergence. Clearly, further studies in this area are essential, as the therapeutic options for enteric fever continue to narrow. Currently, third-generation cephalosporins or fluoroquinolones are the only real options available for enteric fever infections that are MDR and nonsusceptible to ciprofloxacin, yet increasing reports of resistance with these agents mean that azithromycin may itself emerge as a crucial drug in the future treatment and control of enteric fever.

## References

[1] Buckle GC, Fischer Walker CL, Black RE 2012 Typhoid fever and paratyphoid fever: systematic review to estimate global morbidity and mortality for 2010. J Glob Health 2(1):010401. doi:10.7189/jogh.02.010401. 23198130 PMC3484760

[2] Butler T 2011 Treatment of typhoid fever in the 21st century: promises and shortcomings. Clin Microbiol Infect 17:959–963. doi:10.1111/j.1469-0691.2011.03552.x. 21722249

[3] Chau TT, Campbell JI, Galindo CM, Hoang NVM, Diep TS, Nga TT, Chau NVV, Tuan PQ, Page AL, Ochiai RL, Schultsz C, Wain J, Bhutta ZA, Parry CM, Bhattacharya SK, Dutta S, Agtini M, Dong B, Honghui Y, Anh DD, Do GC, Naheed A, Albert MJ, Phetsouvanh R, Newton PN, Basnyat B, Arjyal A, La TT, Rang NN, Phuong LT, Bay PVB, von Seidlein L, Dougan G, Clemens JD, Vinh H, Hien TT, Chinh NT, Acosta CJ, Farrar J, Dolecek C 2007 Antimicrobial drug resistance of Salmonella serovar Typhi in Asia and molecular mechanism of reduced susceptibility to the fluoroquinolones. Antimicrob Agents Chemother 51:4315–4323. doi:10.1128/AAC.00294-07. 17908946 PMC2167998

[4] Gaind R, Paglietti B, Murgia M, Dawar R, Uzzau S, Cappuccinelli P, Deb M, Aggarwal P, Rubino S 2006 Molecular characterization of ciprofloxacin-resistant Salmonella serovar Typhi and Paratyphi A causing enteric fever in India. J Antimicrob Chemother 58:1139–1144. doi:10.1093/jac/dkl391. 17071955

[5] Kariuki S, Revathi G, Kiiru J, Mengo DM, Mwituria J, Muyodi J, Munyalo A, Teo YY, Holt K, Kingsley RA, Dougan G 2010 Typhoid in Kenya is associated with a dominant multidrug-resistant Salmonella serovar Typhi haplotype that is also widespread in Southeast Asia. J Clin Microbiol 48:2171–2176. doi:10.1128/JCM.01983-09. 20392916 PMC2884483

[6] Keddy KH, Smith AM, Sooka A, Ismail H, Oliver S 2010 Fluoroquinolone-resistant typhoid, South Africa. Emerg Infect Dis 16:879–880. doi:10.3201/eid1605.091917. 20409393 PMC2954527

[7] Al Naiemi N, Zwart B, Rijnsburger MC, Roosendaal R, Debets-Ossenkopp YJ, Mulder JA, Fijen CA, Maten W, Vandenbroucke-Grauls CM, Savelkoul PH 2008 Extended-spectrum-beta-lactamase production in a Salmonella enterica serotype Typhi strain from the Philippines. J Clin Microbiol 46:2794–2795. doi:10.1128/JCM.00676-08. 18550740 PMC2519485

[8] Butler T, Sridhar CB, Daga MK, Pathak K, Pandit RB, Khakhria R, Potkar CN, Zelasky MT, Johnson RB 1999 Treatment of typhoid fever with azithromycin versus chloramphenicol in a randomized multicentre trial in India. J Antimicrob Chemother 44:243–250. doi:10.1093/jac/44.2.243. 10473232

[9] Girgis NI, Butler T, Frenck RW, Sultan Y, Brown FM, Tribble D, Khakhria R 1999 Azithromycin versus ciprofloxacin for treatment of uncomplicated typhoid fever in a randomized trial in Egypt that included patients with multidrug resistance. Antimicrob Agents Chemother 43:1441–1444. 10348767 10.1128/aac.43.6.1441 PMC89293

[10] Chinh NT, Parry CM, Ly NT, Ha HD, Thong MX, Diep TS, Wain J, White NJ, Farrar JJ 2000 A randomised controlled comparison of azithromycin and ofloxacin for treatment of multidrug-resistant or nalidixic acid resistant enteric fever. Antimicrob Agents Chemother 44:1855–1859. doi:10.1128/AAC.44.7.1855-1859.2000. 10858343 PMC89974

[11] Frenck RW, Nakhla IA, Sultan Y, Bassily SB, Girgis FY, David J, Butler TC, Girgis NI, Morsy M 2000 Azithromycin versus ceftriaxone for the treatment of uncomplicated typhoid fever in children. Clin Infect Dis 31:1134–1138. doi:10.1086/317450. 11073741

[12] Frenck RW, Mansour A, Nakhla I, Sultan Y, Putnam S, Wierzba T, Morsy M, Knirsch C 2004 Short-course azithromycin for the treatment of uncomplicated typhoid fever in children and adolescents. Clin Infect Dis 38:951–957. doi:10.1086/382359. 15034826

[13] Parry CM, Ho VA, Phuong LT, Bay PVB, Lanh MN, Tung LT, Tham NTH, Wain J, Hien TT, Farrar JJ 2007 Randomized controlled comparison of ofloxacin, azithromycin and an ofloxacin-azithromycin combination for treatment of multidrug-resistant and nalidixic acid-resistant typhoid fever. Antimicrob Agents Chemother 51:819–825. doi:10.1128/AAC.00447-06. 17145784 PMC1803150

[14] Dolecek C, La TTP, Rang NN, Phuong LT, Vinh H, Tuan PQ, Du DC, Bay NTB, Long DT, Ha LB, Binh NT, Hong NTA, Dung PN, Lanh MN, Bay PVB, Ho VA, Hoang NVM, Nga TTT, Chau TT, Shultsz C, Dunstan SJ, Stepniewska K, Campbell JI, Diep TS, Basnyat B, Chau NVV, Sach NV, Chinh NT, Hien TT, Farrar J 2008 A multi-center randomised controlled trial of gatifloxacin versus azithromycin for the treatment of uncomplicated typhoid fever in children and adults in Vietnam. PLoS One 3:e2188. doi:10.1371/journal.pone.0002188. 18493312 PMC2374894

[15] Clinical and Laboratory Standards Institute. 2013 Performance standards for antimicrobial susceptibility testing; 23rd informational supplement. CLSI document M100-S23. Clinical and Laboratory Standards Institute, Wayne, PA.

[16] British Society for Antimicrobial Chemotherapy. 2014 BSAC methods for antimicrobial susceptibility testing. Version 13, May 2014. http://bsac.org.uk/susceptibility/methodologylatestversion/.

[17] European Committee on Antimicrobial Susceptibility Testing. 2014 Breakpoint tables for interpretation of MICs and zone diameters. Version 4.0, January 2014. http://www.eucast.org/clinical_breakpoints/.

[18] Clinical and Laboratory Standards Institute. 2012 Methods for dilution antimicrobial susceptibility tests for bacteria that grow aerobically; approved standard, 9th ed CLSI document M07-A9. Clinical and Laboratory Standards Institute, Wayne, PA.

[19] Phuc Nguyen MC, Woerther PL, Bouvet M, Andremont A, Leclercq R, Canu A 2009 Escherichia coli as reservoir for macrolide resistance genes. Emerg Infect Dis 15:1648–1650. doi:10.3201/eid1510.090696. 19861064 PMC2866414

[20] Metzler DM, DeHaan RM 1974 Susceptibility tests of anaerobic bacteria: statistical and clinical considerations. J Infect Dis 130:588–594. doi:10.1093/infdis/130.6.588. 4372272

[21] Clinical and Laboratory Standards Institute. 2011 Development of *in vitro* susceptibility testing criteria and quality control parameters; approved guideline, 3rd ed CLSI document M23-A3. Clinical and Laboratory Standards Institute, Wayne, PA.

[22] Fernando S, Molland JG, Gottlieb T 2012 Failure of oral antibiotic therapy, including azithromycin, in the treatment of a recurrent breast abscess due to Salmonella enterica serotype Paratyphi A. Pathog Glob Health 106:366–369. doi:10.1179/2047773212Y.0000000010. 23182142 PMC4005136

[23] Molloy A, Nair S, Cooke FJ, Wain J, Farrington M, Lehner PJ, Torok ME 2010 First report of Salmonella enterica serotype Paratyphi A azithromycin resistance leading to treatment failure. J Clin Microbiol 48:4655–4657. doi:10.1128/JCM.00648-10. 20943875 PMC3008474

[24] Gunell M, Kotilainen P, Jalava J, Huovinen P, Siitonen A, Hakanen AJ 2010 *In vitro* activity of azithromycin against nontyphoidal Salmonella enterica. Antimicrob Agents Chemother 54:3498–3501. doi:10.1128/AAC.01678-09. 20498312 PMC2916314

[25] Sjölund-Karlsson M, Joyce K, Blickenstaff K, Bell T, Haro J, Medalla FM, Fedorka-Cray P, Zhao S, Crump JA, Whichard JM 2011 Antimicrobial susceptibility to azithromycin among Salmonella enterica isolates from the United States. Antimicrob Agents Chemother 55:3985–3989. doi:10.1128/AAC.00590-11. 21690279 PMC3165283

[26] Dutta S, Das S, Mitra U, Jain P, Roy I, Ganguly SS, Ray U, Dutta P, Paul DK 2014 Antimicrobial resistance, virulence profiles and molecular subtypes of Salmonella enterica serovars Typhi and Paratyphi A blood isolates from Kolkata, India during 2009-2013. PLoS One 9:e101347. doi:10.1371/journal.pone.0101347. 25098613 PMC4123848

[27] Butler TC, Frenck RW, Johnson RB, Khakhria R 2001 *In vitro* effects of azithromycin on Salmonella typhi: early inhibition by concentrations less than the MIC and reduction of MIC by alkaline pH and small inocula. J Antimicrob Chemother 47:455–458. doi:10.1093/jac/47.4.455. 11266420

[28] Foulds G, Shepard RM, Johnson RB 1990 The pharmacokinetics of azithromycin in human serum and tissues. J Antimicrob Chemother 25(Suppl A):73–82. doi:10.1093/jac/25.suppl_A.73. 2154441

[29] Panteix G, Guillaumond B, Harf R, Desbos A, Sapin V, Leclercq M, Perrin-Fayolle M 1993 In-vitro concentration of azithromycin in human phagocytic cells. J Antimicrob Chemother 31(Suppl E):1–4. doi:10.1093/jac/31.suppl_E.1. 8396080

[30] Rakita RM, Jaques-Palaz K, Murray BE 1994 Intracellular activity of azithromycin against bacterial enteric pathogens. Antimicrob Agents Chemother 38:1915–1921. doi:10.1128/AAC.38.9.1915. 7810998 PMC284662

[31] Van Bambeke F, Tulkens PM 2001 Macrolides: pharmacokinetics and pharmacodynamics. Int J Antimicrob Agents 18:S17–S23. doi:10.1016/S0924-8579(01)00406-X. 11574190

[32] Kobayashi T, Hayakawa K, Mawatari M, Mezaki K, Takeshita N, Kutsuna S, Fujiya Y, Kanagawa S, Ohmagari N, Kato Y, Morita M 2014 Case report: failure under azithromycin treatment in a case of bacteremia due to Salmonella enterica Paratyphi A. BMC Infect Dis 14:404. doi:10.1186/1471-2334-14-404. 25041573 PMC4223471

[33] Hassing RJ, Goessens WHF, van Pelt W, Mevius DJ, Stricker BH, Molhoek N, Verbon A, van Genderen PJJ 2014 Salmonella subtypes with increased MICs for azithromycin in travellers returned to the Netherlands. Emerg Infect Dis 20:705–708. doi:10.3201/eid2004.131536. 24655478 PMC3966360

[34] Rai S, Jain S, Prasad KN, Ghoshal U, Dhole TN 2012 Rationale of azithromycin prescribing practices for enteric fever in India. Indian J Med Microbiol 30:30–33. doi:10.4103/0255-0857.93017. 22361757

